# The impact of later eating rhythm on childhood adiposity: protocol for a systematic review

**DOI:** 10.1186/s13643-019-1226-y

**Published:** 2019-11-26

**Authors:** Mengxuan Zou, Kate Northstone, Rachel Perry, Laura Johnson, Sam Leary

**Affiliations:** 1 Bristol Dental School, Faculty of Health Sciences, University of Bristol, Bristol, BS2 8AE UK; 2 Bristol Medical School, Faculty of Health Sciences, University of Bristol, Bristol, BS8 2BN UK; 3 NIHR Biomedical Research Centre in Nutrition, University of Bristol, Bristol, BS2 8AE UK; 4 Centre for Exercise, Nutrition and Health Sciences, School of Policy Studies, University of Bristol, Bristol, BS8 1TZ UK

**Keywords:** Adiposity, Obesity, Childhood, School age children, Adolescence, Night eating, Later eating rhythm

## Abstract

**Background:**

Childhood adiposity has increased dramatically in the last few decades and is an important predictor of adulthood chronic disease. Later eating rhythm, termed night eating (NE), is increasingly prevalent in adults; however, the prevalence of NE in children and relationship between NE and adiposity in children still remains uncertain. The aim of this work is to review the association between adiposity in children and adolescents and NE, in terms of calorie intake, timing and meal frequency in the evening/night.

**Methods:**

The Cochrane library, CINAHL, Embase, MEDLINE (via OVID) and Web of Science databases will be searched from inception to November 2019 for randomised controlled trials (RCTs) and observational studies (cohort, cross-sectional and case-control studies) which investigate the association between later vs. earlier timing of food intake at night or relatively more vs. less energy intake in any eating occasions or time period after 4 pm on adiposity in children and adolescents (4–18 years). The outcomes will be body mass index (BMI)/BMI standard deviation score (BMI-SDS or BMI *Z*-score), waist circumference (WC), fat mass index (FMI)/percentage of body fat (%BF) or waist to hip ratio (WHR). No language restriction will be applied. Screening for eligibility from the title and abstracts and data extraction from the full texts will be carried out by two reviewers independently. References listed in the included studies will be hand-searched for any additional articles. The quality of included RCT studies will be assessed using Revised Cochrane Risk of Bias tool (RoB 2), and of observational studies using Newcastle Ottawa scale. A qualitative synthesis of the results will be presented, and meta-analysis will be conducted, where appropriate.

**Discussion:**

The planned systematic review will investigate the association between later eating rhythm and adiposity in children and adolescents. Understanding the best meal size, timing of energy intake and meal frequency across the evening time for maintaining healthy weight in children is important in order to give parents the best advice to help prevent adulthood obesity and associated chronic diseases in their children.

**Systematic review registration:**

PROSPERO CRD42019134187.

## Background

Although the term obesity is generally used in English, the Latin term adiposity has broader meaning, encompassing both obesity (excess fat mass) and the fat in fatty tissue [1]. In addition, more anthropometric measurements, such as BMI, WC, FMI and WHR, have been applied to assess adiposity compared with obesity, which is commonly derived from categorized BMI [2]. Childhood adiposity is an important predictor of adulthood obesity and its related comorbidities including type 2 diabetes and cardiovascular disease [3, 4]. Globally, the prevalence of obesity among children and adolescents aged from 5 to 19 years old has increased from 0.7% in 1975 to 5.6% in 2016 in girls and from 0.9% in 1975 to 7.8% in 2016 among boys [5]. Besides, in preschool-age children, the prevalence of overweight and obesity is expected to reach 9.1% or around 60 million by 2020 [6].

The identification of risk factors for adiposity is the key for prevention. Four key modifiable lifestyle behaviours have been identified as influencing body weight among children: physical activity, sleep, screen time and eating habits [7]. In particular, developing healthy eating habits early in life is a way to prevent the onset of adulthood diet-related diseases, and the dietary causes of adiposity are complex [8, 9]. Recent studies have linked energy regulation to the circadian clock at the behavioural, physiological and molecular levels, emphasizing that the timing of food intake itself may play a significant role in adiposity development [10]. Many studies have recognized the importance of circadian rhythm in regulating human food intake behaviour and metabolism [11]. For example, the transport of lipids [12], glucose [13] and dietary protein [14] in the intestine has been shown to be involved in a circadian-driven process. Meanwhile, it is commonly accepted that nocturnal eating can result in metabolic disruption; research has suggested that energy metabolism is less efficient during the evening [15, 16]. Food and beverages consumed in the evening tend to be more energy dense, with dinner typically being the most energy-dense main meal of the day in most high-income countries [17, 18]. However, evidence on the effects of eating habits during the evening and night for childhood adiposity is currently limited.

The association between NE and childhood adiposity can be implied from the impact of breakfast consumption on childhood adiposity based on the relationship between breakfast and dinner consumption. Proverbs such as ‘breakfast like a king, lunch like a prince and dinner like a pauper’ or ‘eat breakfast yourself, share lunch with a friend and give dinner away to your enemy’ which recommend a reduction of energy intake across the day remain commonly held beliefs of healthy dietary recommendation and weight management [19]. However, no recommendation for the optimal distribution of energy intake across the day is available so far. Studies have confirmed the positive association of breakfast skipping on childhood obesity [20–22]. Interestingly, evidence showed that overweight/obesity in breakfast skippers has been related to a higher energy and carbohydrate intake at dinner [23], meaning that the association between breakfast skipping and obesity may relate to higher energy intake in the evening or night due to calorie deficit from breakfast. However, the direct relationship between food intake during evening and night and childhood obesity remains uncertain.

Previous studies concentrating on food intake during the evening and night in children tended to term later eating pattern as ‘night eating’ (NE), which was first described as a specific behaviour pattern more than 50 years ago [24]. No consistent definition has been agreed since then [25–31]. However, NE is generally accepted to be defined by three aspects: time, energy intake and frequency.

To our knowledge, there is only limited evidence illustrating how energy intake is distributed over the day especially at night and its potential relationship with obesity in children. A review of current evidence from eleven observational studies on global trends in time-of-day of energy intake in Western, Eastern, Northern and Southern Europe, and in North and South America found that the energy distribution over the day varied by countries and geographical area [32]. Only one study demonstrated a trend towards increased energy intake later in the day in British adults between 1982 and 1999 [33]; however, no evidence in children was found due to limited data of secular trends in children. In relation to adiposity, four cross-sectional studies and one longitudinal cohort were included; in this review, only two of them showed positive association between energy intake at evening/night and BMI (body mass index) in children aged 4–12 years old. However, there is a wide variation in the assessment of diet and weight status in these studies. A recent systematic review included ten observational studies investigating the association between large dinner and excess weight in adults; four studies showed a positive association; five studies showed no association, and one showed an inverse relationship. The meta-analysis of observational studies did not show an association between evening eating and BMI (*P* = 0.06) [17]. However, this study was limited by its significant heterogeneity and exclusion of children.

The objective of this review will be to evaluate the association between adiposity in children and adolescents and later eating rhythm in terms of calorie intake, timing and frequency of meal consumption in the evening.

## Methods

This protocol has been reported in accordance with the Preferred Reporting Items for Systematic Reviews and Meta-Analyses for Protocols (PRISMA-P) 2015 reporting guideline [34] (see Additional file 1).

### Eligibility criteria

To be included in this review, studies must meet the following criteria.

#### Study design

Randomized controlled trials (RCTs), observational study designs such as cohort studies, cross-sectional studies and case-control studies will be included. Only original research studies will be included. Review, case studies and survey will be excluded, but the references of review articles will be searched for further studies. The results from abstracts and conference papers will be considered and narratively synthesized where appropriate.

#### Participants

Studies involving participants aged 4–18 years. NE in adults is heavily associated with occupation and night eating syndrome (NES), and adult only studies will be excluded. In addition, studies involving both children and adults that did not stratify by age group will be excluded. Studies involving the critically ill or those with endocrine disorders or syndromic obesity will be excluded.

#### Intervention/exposure

The definition of NE is likely to differ between studies, especially between countries due to cultural differences in the timing of eating occasions. NE is generally described from three aspects: timing, energy intake and meal frequency; the intervention/exposure will be classified as follows:

- From the perspective of timing: later meal or snack time in the evening (4–11.59 pm). Studies need to exhibit the timing or time period of meals or snacks in the evening (4–11.59 pm).
- From the perspective of calories intake: diet where a greater proportion of total daily energy intake (TDEI) is consumed in the evening (4–11.59 pm). Studies need to quantify the proportion of TDEI in the evening time period or the published data allow for its calculation.
- From the perspective of eating occasions: more meal/ snack/drink occasions in the evening (4–11.59 pm).
- For those studies which define NE utilizing same criteria in terms of timing, calories intake and frequency of NE, the intervention/exposure would consistently be NE. For example, the intervention/exposure of those studies which define NE using the same criteria that consuming more than 25% of daily energy intake after 7 pm in all recording days will be NE.
- For those studies which define NE utilizing different criteria in terms of either timing or calories intake or frequency of NE, this review will organize article results by indicating the difference between exposure/intervention and comparison groups. For example, studies may define NE as consuming more than 25% of daily energy intake after last meal time, other studies may define NE as consuming more than 20% of daily energy intake after 7pm. The difference of criteria between studies will be indicated when organizing.

Regarding on dietary assessment, articles using measurements such as 24hr food recall with at least one recorded day, food diary with at least one recorded day, direct observation and food frequency questionnaire will be included.

#### Comparison

The comparison will be classified as followed in accordance with intervention/exposure:

- From the perspective of timing: earlier meal or snack time in the evening.
- From the perspective of calories intake: diet where a smaller proportion of TDEI is consumed in the evening (4–11.59 pm).
- From the perspective of eating occasions: fewer meal/ snack/drink occasions.
- For those studies which define NE utilizing the same criteria (specifically from timing, calories intake and frequency of NE during recording food diaries): non-NE.

#### Outcomes

To be included, studies must report at least one of the following measurements of childhood adiposity:

- BMI/BMI-SDS/BMI *Z*-score
- WC
- FMI/%BF
- WHR

#### Language

No restrictions will be applied for language. Non-English articles will be translated where possible.

### Information sources and search strategy

Literature search strategies will be developed and checked by an experienced systematic reviewer (RP) in the following electronic databases for relevant published articles:

- Embase (from 1974 to present)
- MEDLINE (from 1946 to present)
- Web of Science (from 1900 to present)
- Cochrane library (from inception to present)
- CINAHL (from 1937 to present)

An example of the search strategy for use in MEDLINE is shown in Additional file 2. The search strategy for each database will be similar but revised appropriately to take into account any differences in controlled vocabulary and syntax rules.

Published systematic reviews will not be included in this review but will be used to identify further articles not found in the original search. Reference lists of all included articles as well as review articles will be hand searched for additional original articles. Searches will be updated every 6 months to identify any further articles since the initial search. Conference papers and abstracts will be used to help identify potential articles, and authors will be contacted to see if full-text articles are available.

### Study records

#### Data management

The EndNote reference management software package will be used to manage all the search results throughout the process of this review. Database search results will be imported into an EndNote library and duplicates will be removed during the data screening process (MZ, RP).

#### Selection process

Titles and abstracts will be screened for eligibility independently by the first reviewer (MZ) and other members of the review team (KN, RP, LJ, SL). Articles with titles/abstracts meeting the inclusion criteria will be retrieved in full for further screening. The full texts of potentially relevant studies (from the reference list of included articles) or articles from other sources known to authors will also be retrieved and screened by the same two reviewers (MZ, RP) according to the inclusion and exclusion criteria. Any disagreement between the two reviewers will be discussed with a third reviewer (KN) for resolution; the reasons for excluding studies will be recorded.

Data collection process

The full texts of all the included articles will be retrieved, and data will be extracted independently by four reviewers according to a standardised data extraction template developed specifically for this review. The first reviewer (MZ) will extract all the included articles; the other reviewer (SL, KN and RP) will do the double extraction, one-third of all articles each. The template will be piloted by all reviewers (SL, KN and RP) on the extraction of the first three papers, then the reviewers will discuss to ensure common use and to amend as required to ensure consistency.

#### Data extraction

The following information will be extracted from each article:

- General information:
  - Author (year of publication)
  - Year the study took place
  - Study funding sources
  - Possible conflicts of interests
  - Ethical approval obtained for study.
- Study eligibility:
  - Type of study (e.g. RCT, cohort study, cross sectional study, case control study, abstract/conference paper)
  - Participants: sex, age, race, location, setting, sample size, socioeconomic position (e.g. parental income, education, residential area etc.), other demographic factors
  - Exposure variable, dietary assessment method
  - Outcome variable, outcome assessment method
- Results:
  - Prevalence of exposure
  - Prevalence of outcome
  - Effect sizes: dichotomous outcomes (e.g. odds ratio, relative risk, hazard ratio) or continuous outcomes (e.g. mean difference, standardized mean difference), all with 95% CIs and *p* values
- Analysis methods

Any disagreements found will be discussed with a third reviewer (KN) in order to finalise data extraction.

#### Quality assessment (risk of bias in individual studies)

The full texts of all included articles will be assessed for methodological quality and risk of bias by two reviewers independently. Any discrepancies in study quality assessment between the two reviewers will be resolved through discussion with the third reviewer. RCTs will be assessed using the Revised Cochrane Risk of Bias tool (RoB 2) [35]. Studies will be classified as having a low risk of bias, high risk of bias or some concerns based on five bias domains:

- Bias arising from randomisation process
- Bias due to deviations from intended interventions
- Bias due to missing outcome data
- Bias in measurement of the outcome
- Bias in selection of the reported result

Within each domain, one or more signalling questions will be asked. Observational studies will be assessed for risk of bias by using Newcastle Ottawa scale [36]. A ‘star system’ has been developed in which the quality of studies will be assessed from three perspectives:

- Selection of the study groups
- Comparability of the study groups
- Ascertainment of either the exposure or outcome of interest

Harvest plots of effect estimates stratified according to individual study quality will be used to examine the potential for bias to influence the results of studies where appropriate [37].

### Data synthesis

This study will present a narrative synthesis of the findings of all included articles. Stata 15.0 will be used for meta-analyses where needed. Owing to the diverse nature of the interests of articles, heterogeneity between articles is anticipated, and therefore, random effects models will be utilised to quantitatively synthesise all data where possible. Heterogeneity between articles will be assessed by visually inspecting forest plots and with chi-square measurement in which the cutoff of *P* < 0.10 will be set to indicate heterogeneity since it is difficult to assess when sample size are small. The *I*^2^ statistic will be used to measure variation in the effect size due to heterogeneity, with values greater than 50% indicative of significant heterogeneity [38]. In case of significant heterogeneity, subgroup analyses will be conducted (see ‘Sub-group analyses’ for further detail). We will test the likelihood of publication bias through visual inspection of funnel plots and using Egger’s regression test which would be presented when there are at least 10 studies included in the meta-analysis [39]. If a meta-analysis is undertaken, the GRADE system will be used to assess the strength of the body of evidence [40].

#### Sub-group analyses

Results of observational studies and RCTs will be meta-analysed, separately. Considering that eating pattern and measurement for obesity are significantly different between children and adolescents, results will be synthesised by age group (e.g. preschool-age, young children, older children, adolescents) separately. If the articles are considered too heterogeneous in term of the non-comparable exposure/outcome measurements, exposure/outcome definition or intervention format or populations, articles will further be synthesised separately by gender, socio-economic status, geographical area, different assessment of food intake (questionnaire vs detailed multi-day food diary) and types of exposure such as timing of meal intake in the evening, proportion of TDEI consuming in the evening/night or specific time period, the frequency of NE in the recorded days and the frequency of meal in the evening/night. The narrative synthesis of the findings will be presented, and meta-analysis result will be exhibited when there are sufficient articles in the sub-group.

#### Sensitivity analyses

If a meta-analysis is undertaken, any factors such as methodological differences (data collection and statistical analyses) or articles at high risk of bias or with imputation of missing data may significantly affect the pooled estimates; therefore, sensitivity analyses will be conducted to explore the impact of the above factors [38]. For example, the prevalence of NE may differ due to different measurement of food intake, such as 24-h recall food diary and multi-day food diary, main analyses will be repeated by excluding studies using 24-h recall food diary which may less likely to define habitual behaviour. In terms of missing data, the meta-analyses will be repeated by excluding studies in which missing data were imputed.

## Discussion

There are several strengths of this protocol. RCTs will be assessed using the latest Cochrane risk of bias tool for RCTs (RoB 2) which has been revised based on the original RoB tool by addressing previous issues, incorporating advances in other recently developed tools and integrating recent developments in estimation of intervention effects. Considering the various definition of exposure (e.g. timing, calories intake or meal frequency), we plan to synthesis the articles by type of exposures, by which we can review the association between later eating rhythm and childhood adiposity from different aspects separately. Limitations of this protocol are also anticipated. According to the literature pre-searching, we did not find any RCTs, and the majority of studies were cross sectional. While cross-sectional studies are unable to establish causal associations, we still feel that these types of studies should be included in the review; otherwise, a large proportion of the literature would be omitted. We will perform subgroup analysis by study types and assess the risk of bias using modified Newcastle Ottawa scale for cross sectional studies.

To our knowledge, this will be the first systematic review investigating the association between night eating behaviour and adiposity in children and adolescents. There is a common belief that ‘breakfast like a king, lunch like a prince and dinner like a pauper’ is an appropriate dietary recommendation; however, the evidence-based recommendation of energy distribution across the day is still unavailable so far, especially, the role of later eating rhythm is still poorly understood. Besides, eating habit has been shown to develop early in childhood and track further on until adulthood, and healthy eating habits early in life is a way to prevent the onset of diet-related disease. To our knowledge, no systematic review has studied childhood later eating rhythm and its effect on adiposity. Furthermore, considering the recognized physiologically effect of NE on adiposity, this study is urgently needed. We therefore believe that the findings will fill gaps in knowledge about the role of childhood later eating rhythm on obesity and help parents understand the best meal size, timing of energy intake and meal frequency across the evening for maintaining healthy weight in children, thereby helping them prevent adulthood obesity and associated chronic diseases in their children.

Any substantive amendments to this protocol will be registered with PROSPERO as they occur and documented in the final publication.

## Dissemination

We will publish review results in an international peer-reviewed journal and will report results based on the statement in the Preferred Reporting Items for Systematic Reviews and Meta-Analyses (PRISMA). Besides, we will disseminate results to the research community and relevant stakeholders through presentations at relevant academic conference.

## Supplementary information

**Additional file 1.** PRISMA-P (Preferred Reporting Items for Systematic review and Meta-Analysis Protocols) 2015 checklist.

**Additional file 2.** An example of the search terms used in MEDLINE.

## Abbreviations

%BF: Percentage of body fat
BMI: Body mass index
BMI-SDS: BMI standard deviation score
FMI: Fat mass index
HR: Hazard ratio
MD: Mean difference
NE: Night eating
NES: Night eating syndrome;
OR: Odds ratio
RCT: Randomised controlled trial
RR: Relative risk
SMD: Standardized mean difference
TDEI: Total daily energy intake
WC: Waist circumference
WHR: Waist to hip ratio
