# Supplementary material for: The impact of later eating rhythm on childhood adiposity: protocol for a systematic review
Source: Syst Rev. 2019 Nov 26;8:290. doi: 10.1186/s13643-019-1226-y (PMC6878614; doi:10.1186/s13643-019-1226-y)
Supplement: Supplementary file 2 — Additional file 2. An example of the search terms used in MEDLINE. [file 13643_2019_1226_MOESM2_ESM.docx]

**ADDITIONAL FILE 2: MEDLINE SEARCH TERMS:**

1. exp CHILD/

2. juvenile.tw.

3. exp INFANT/

4. exp PEDIATRICS/

5. child*.tw.

6. infant*.tw.

7. teen*.tw.

8. p?ediatric*.tw.

9. adolescen*.tw.

10. (young adj1 person*).tw.

11. schoolchild*.tw.

12. youth.tw.

13. (boy* or girl*).tw.

14. 1 or 2 or 3 or 4 or 5 or 6 or 7 or 8 or 9 or 10 or 11 or 12 or 13

15. over?eat*.tw.

16. (eat* adj1 late).tw.

17. (night* adj1 eat*).tw.

18. (time adj1 to adj1 eat*).tw.

19. (time adj1 eat*).ti,ab.

20. dinner.tw.

21. ((night or late or evening) adj1 (eat* or snack* or meal or dinner)).tw.

22. (eating adj1 behavio?r*).tw.

23. night eating.ti,ab.

24. nocturnal eating.ti,ab.

25. 15 or 16 or 17 or 18 or 19 or 20 or 21 or 22 or 23 or 24

26. BMI.tw.

27. adiposity.tw.

28. waist.tw.

29. hip.tw.

30. (body fat or body fat percent* or percent* body fat or fat mass or adipos*).ti,ab.

31. (body adj weight).tw.

32. obes*.tw.

33. overweight.tw.

34. 26 or 27 or 28 or 29 or 30 or 31 or 32 or 33

35. (cross adj1 sectional).tw.

36. trial.tw.

37. ((cohort or inciden* or prospective or follow-up or longitudinal or case-control or case) adj1 control).tw.

38. survey.tw.

39. quasi experimental study/ or experimental study/ or prospective study/ or cohort study/

40. observational study/ or exp clinical study/ or study/ or longitudinal study/

41. exp case control study/ or population based case control study/ or "multicenter study (topic)"/ or feasibility study/ or exp cross-sectional study/

42. exp conference paper/

43. 35 or 36 or 37 or 38 or 39 or 40 or 41 or 42

44. 14 and 25 and 34 and 43

45. animals/ not humans/

46. exp rodent/

47. (rat or rats or mouse or mice).tw.

48. 45 or 46 or 47

49. 44 not 48
